# Applying Progress Testing to Undergraduate Periodontal Courses—A First Evaluation After 2 Years of Use in a German University

**DOI:** 10.1111/eje.70073

**Published:** 2025-10-31

**Authors:** Anna Louisa Kollster, Miriam Cyris, Jonas Conrad, Mohamed Mekhemar, Christof E. Dörfer, Christian Graetz

**Affiliations:** ^1^ Clinic of Conservative Dentistry and Periodontology University Hospital of Schleswig‐Holstein Kiel Germany

**Keywords:** eriodontology, progress test, teaching programme, undergraduate

## Abstract

**Background:**

The Progress Test in Periodontology (PTP) has been implemented at Kiel University to gain an impression of students' learning process in this specific subdiscipline of periodontology within the dental curriculum.

**Methods:**

The PTP has been designed following internationally established standards and has been adapted into the context of periodontology. From July 2020 to February 2022, it has been applied to undergraduate students. The PTP consists of 30 single‐best answer questions including an ‘I don't know’ option, drawn from a newly created question bank and weighted by an adapted blueprint. Descriptive analysis and statistics to test validity and reliability have been performed.

**Results:**

The mean score shows a significant growth over the semesters (42.4% ± 11.7% after the first semester, 61.5% ± 13.8% after the second semester, 76.9% ± 12.8% after the third semester, 69.9% ± 12.7% after the fifth semester). Reliability expressed by Cronbach's *α* ranges from 0.646 to 0.798. The ‘I don't know’ option has mainly been used by first semester students.

**Conclusion:**

Within the limitations of the study, the PTP is a valid and reliable tool to evaluate students' gain of knowledge, even if it is limited to one subject within a curriculum. Due to pandemic restrictions several other challenges in the adaptation of the test format to the educational setting at a German university had to be solved.

## 1 Introduction

Evaluation of teaching is very important to assess and improve study programmes—especially in subdisciplines of medicine such as dentistry, as graduates carry high responsibilities as physicians [1]. However, it is crucial to consider and choose an appropriate evaluation approach and setting in order to obtain meaningful results [2] accompanying curricular changes and interventions [3]. To overcome this complexity, the progress test (PT) as an outcome‐based test method focuses on the acquirement of knowledge as an attribute of teaching success and therefore helps to conveniently gain an impression of the students' learning progress [4]. PT is aimed at obtaining quantified information about longitudinal knowledge growth and comparison within cohorts, providing feedback for teachers and students as an instrument for evaluation [5, 6]. It encourages students to revise regularly and therefore improves long‐term knowledge retention [7, 8]. The main characteristic of PT is that the same test is administered by students of all levels and calibrated by sampled competences expected from a graduate student. The test administration is repeated regularly in a defined time interval. Each test consists of multiple‐choice questions sampled from a collected item bank. At last, it allows curriculum‐independent comparison of groups and furthermore feedback on curricular changes [9, 10, 11]. Whereas PT has been developed simultaneously in the 1970s at the University of Maastricht, Netherlands [5] and at the University of Missouri, Kansas City, USA [12] it has been used in medical settings worldwide in undergraduate and postgraduate programmes [13, 14, 15]. There are also dental schools that have implemented PTs [16, 17, 18]. Nevertheless, to our best knowledge, no publication has mentioned a PT in periodontology until this day. Thus, and in order to assess the current periodontal curriculum within the dentistry programme of our university, an evaluation has been targeted. Besides the PT, there are several ways of evaluation, comparable to written examinations, being focused on the outcomes of teaching in a formative setting [1].

Therefore, the study's aim was to analyse the implementation of a PT in periodontal teaching (PTP) at the University of Kiel in order to gain insights into the learning process of undergraduate students and use the results to evaluate the quality of current teaching.

## 2 Materials and Methods

### 2.1 Educational Setting

In Germany, dentistry is a 5‐year study programme divided into a five‐semester preclinical and a five‐semester clinical part. At the University of Kiel, periodontology is taught longitudinally during the first, second and third clinical semesters (The sixth, seventh and eighth semesters in total) in the context of the other areas of conservative dentistry. The curriculum includes weekly lectures and seminars supported by practical courses. Before the introduction of the *progress test in periodontology* (PTP), written exams with mainly multiple‐choice questions have been written at the end of every semester. No periodontal courses are taking place during the fourth and fifth semesters. At the end of the curriculum, an oral/practical exam including periodontology occurs. Therefore, additional seminars are offered previously, repeating and summarising the periodontological contents. Resulting from pandemic restrictions, all lectures were digitalised in spring 2020 and due to asynchronous remote teaching the implementation of the online PT has been established instead of written exams at this time.

### 2.2 Blueprint Construction for Progress Test in Periodontology

Each test includes 30 multiple‐choice questions regarding different aspects of the periodontology curriculum (Table 1). A two‐dimensional blueprint has been created, ensuring to cover every topic and the consistency of the assessment to enhance its comparability over time [15, 19]. Additionally, a blueprint is supposed to increase the test's content validity [20]. The grid structure is primarily adopted to the 2018 periodontal classification [2, 21]. Columns represent a certain skill in every subtype of periodontal disorder. As some topics are more central than others, for example, the therapy of periodontitis, they are weighted according to the number of teaching hours spent on the blueprint area (contact hours) [22].

**TABLE 1:** Blueprint matrix for the progress test based on periodontal teaching. Each number represents the number of items in every test out of a certain blueprint area.

	Anatomical, histological and microbiological basics	Aetiology and prognosis	Diagnostics	Classification and symptoms	Therapy methods
Gingival and periodontal health	1	0	1	0	2
Gingival disease and conditions	1	0	1	0	3
Periodontal disease and conditions	0	3	3	2	8
Specification/Modifications of periodontal disease	0	0	1	1	3

*Note:* Total amount of items = 30.

### 2.3 Question Writing and Review

The test items have been written according to the guide of the German PT in medicine (PTM) in the single best answer format to achieve high validity and reliability [23, 24, 25, 26] (^1^). In the developing process, one investigator (A.L.K.) designed the questions that have been reviewed by all teaching staff in the department of periodontology, adapted from an established procedure [15]. Three to five distractors were used [24] (60% four distractors, 34% five distractors, 6% three distractors), one of them being an ‘I don't know’ option. It has been included in all questions to discourage students from guessing [27, 28] and increase reliability [29]. It has been aimed towards that the students won't be able to easily find the correct answer on the internet, but use their acquired knowledge and skills to solve clinical problems. An item bank of 110 different, blueprint conform multiple choice questions has been developed for the first four test occasions. This variety helped to prevent students from studying past questions only in preparation for the test. Ten questions in total have been reused once. Additionally, the questions have not been officially released after the tests.

### 2.4 Test Implementation

**FIGURE 1:**
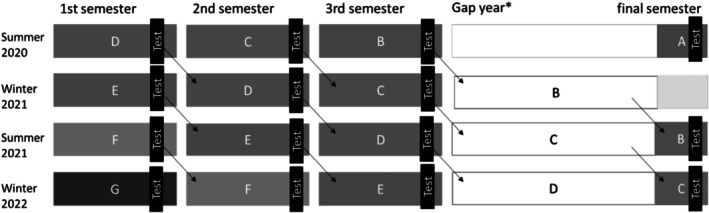
Structure of the cohorts (A–G) participating. *Gap of two semesters without any periodontal courses.

Due to pandemic restrictions and digital teaching, the tests had to be written web‐based at home using the e‐learning platform OpenOlat (frentix GmbH, Zürich, Switzerland), well established at our university. To discourage students from cheating, the test was only formative and had a strict time limit of 25 min, which appeared to be appropriate in pre‐tests. Additionally, the students were not allowed to turn back to questions once answered and could not skip between questions. They have been instructed to use the ‘I don't know’ option instead of guessing if they did not know the correct answer. The test has been set four times at the end of each semester, from July 2020 to February 2022, by the first, second, third clinical and the final semester. A group of students at a certain semester level at a certain test occasion was referred to as a cohort (Figure 1). In semesters 1 to 3, participation was mandatory, but not linked to failing or passing the course. In semester 5, students were offered to take the test as optional feedback for themselves. All data, apart from participation, has been analysed anonymously using an individual identification number. Each correctly answered question has scored one point. Both incorrect or ‘I don't know’ answers were scored neutrally (number right scoring) to avoid bias [27, 30]. No negative marking (subtraction in score in case of wrong answer) has been used as guessing has already been discouraged by the formative setting [28]. The platform allowed no detailed feedback to students; the tool only showed students weak areas by showing the question title used according to the area of the blueprint connected to a check or cross symbol.

### 2.5 Statistical Methods

Test results have been exported from the OLAT platform into SPSS software (SPSS Statistic 28, IBM, Armonk, NY, USA) for statistical analysis. Besides descriptive analysis, results have been undertaken in univariate statistical tests. The Shapiro–Wilk test indicated that the results are not distributed normally. Firstly, the Kruskal–Wallis Test was used to identify significant differences between semesters (*p* < 0.05), corrected by Bonferroni for multivariate testing. Further, we tested validity, assessing to which amount a test measures what it is designed for, and reliability, assessing the reproducibility of results according [20]. Thereby, the construct validity was shown by the measurable growth of knowledge, indicated by the increase of correct answers [12, 13, 31]. Additionally, it was tested through significance testing of the mean results of each semester. Reliability of the test construction has been analysed using internal consistency shown by Cronbach's *α*. Unanswered questions have been supplemented by using the cohort's mean as suggested by Schuwirth et al. [8] and Kuckartz et al. [32] as it was to be expected that students would know the answer to the questions as likely as their peers if they proceeded to it. In order to investigate how well the tests were equated, especially given with a low number of participants and items, the mean item difficulties and standard deviations (SD) between the four test occasions have been compared.

## 3 Results

### 3.1 Overall Results

In total, 397 tests have been registered by 196 individual students (First semester: *n* = 112, second semester: *n* = 120, third semester: *n* = 113, final semester: *n* = 52). Per PT, between 52 and 120 students were enrolled. Seventy percent of all students (*n* = 137) were female (male: *n* = 59 [30%]).

### 3.2 Results of the Progress Test

**FIGURE 2:**
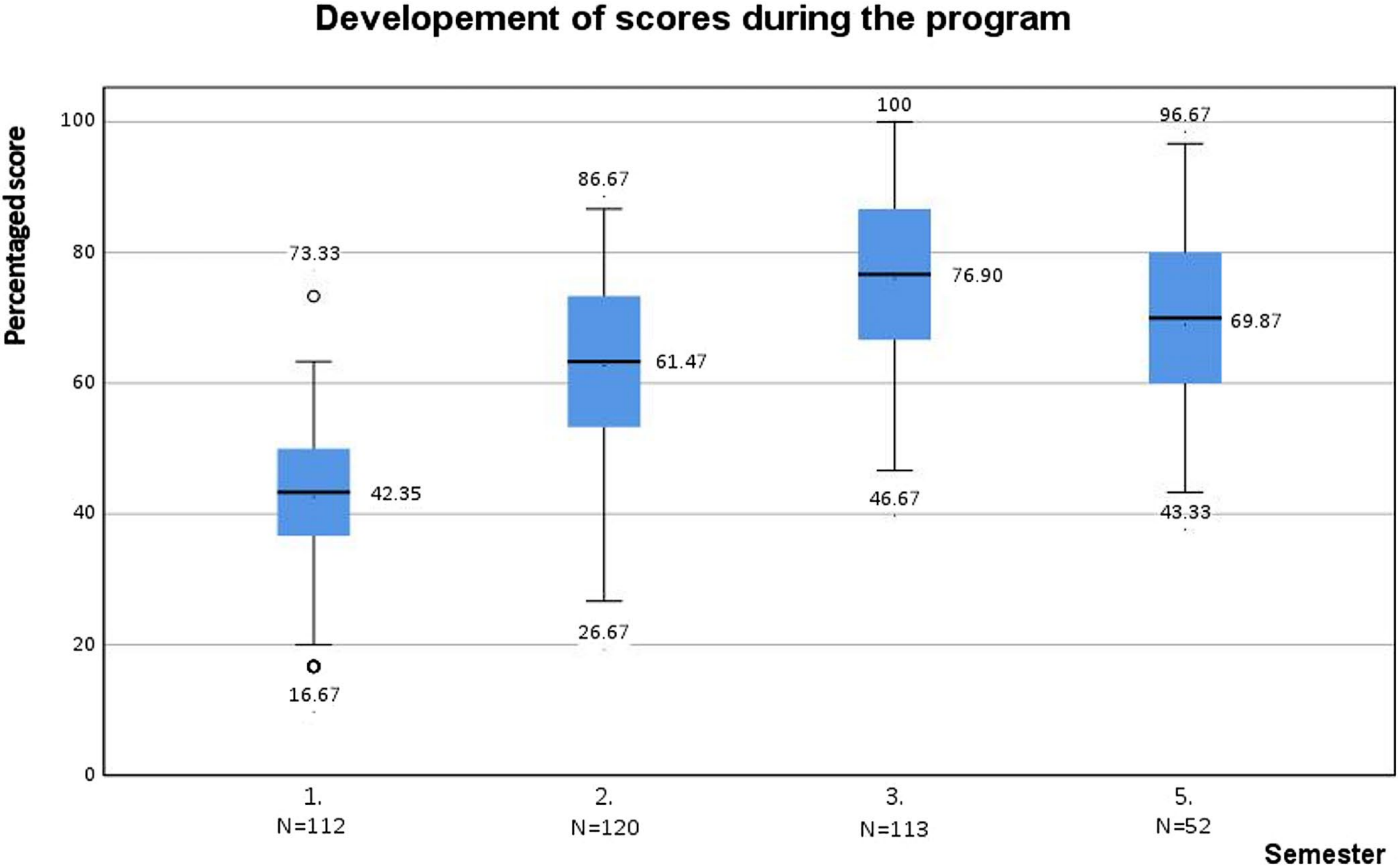
Development of scores (in percent) during the undergraduate curricula periodontology after four semesters, to the target point after the first to third and the fifth semester between July 2020 and January 2022. Pairwise comparison (Kruskal–Wallis test with Bonferroni correction) was significant between all test groups, except between the second and fifth semester (*p* = 0.030) and the third and fifth semester (*p* = 0.129).

The mean score in percent, the correctly answered questions of all asked questions, showed an increase between first, second and third semester and a decrease towards fifth semester (Figure 2). Starting at 42.4% ± 11.7% in first semester, the parameter increased to 61.5% ± 13.8% in second semester, 76.9% ± 12.8% in third semester and finally, after a 1‐year break, to 69.9% ± 12.7% at the end of fifth semester with a significant difference between all semesters (*p* < 0.001). The standard deviation span constantly decreased during the progressing curriculum. Pairwise comparison however indicated that the decrease towards the fifth semester was not significant.

**FIGURE 3:**
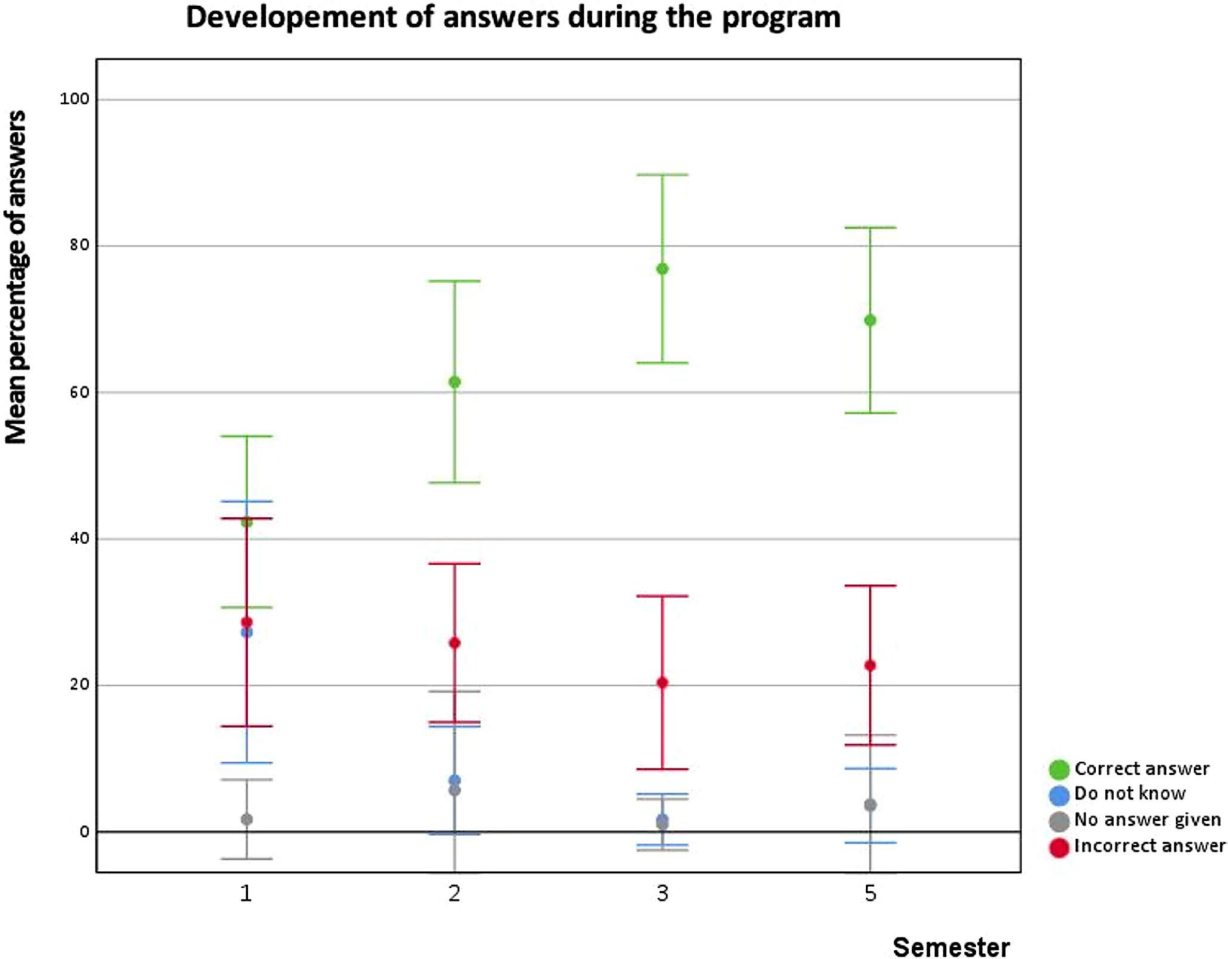
Development of the mean distribution (in percent) of answers according to each semester during the undergraduate curricula periodontology at the four time points between July 2020 and January 2022. Error bars: +/−1 standard deviation.

Figure 3 shows the development of correctly and incorrectly answered questions between the semesters, as well as the use of the ‘I don't know’ option and for various reasons unanswered questions. The ‘I don't know’ option has been used especially by first semester students and decreases significantly between the first and second semesters (*p* < 0.001) and between the second and third semesters (*p* < 0.001). In semesters 3 to 5 it stayed at a level of under 10% and no significant decrease was measurable (*p* = 0.286). Additionally, the number of not given answers neither increased nor decreased during the observed time (*p* = 0.02), whereas between the second and third semesters significant differences happened (*p* = 0.045). Wrong answers were only significantly lower in the third semester compared to the first (*p* < 0.001) and second semesters (*p* = 0.02).

At last, due to a few numbers of participants per test occasion, we found acceptable results of reliability of the test construction calculated by Cronbach's *α* (in total: *α* = 0.6; Table 2). The mean item difficulty of each test varied from 0.58 to 0.677 with a standard deviation between 0.146 and 0.202 (Table 2).

**TABLE 2:** Comparison of test occasions' reliability with Cronbach's *α*.

Test occasion	Total participants	Cronbach's *α*	Mean item difficulty (SD)
1. (Summer 2020)	112	0.798	0.604 (0.202)
2. (Winter 2020/21)	80	0.646	0.580 (0.146)
3. (Summer 2021)	113	0.721	0.602 (0.181)
4. (Winter 2021/22)	92	0.774	0.677 (0.188)

Abbreviation: SD, standard deviation.

## 4 Discussion

This is, to our own knowledge, the first investigation describing the implementation of a Progress Test in an undergraduate curriculum of periodontology worldwide. Driven by the aim of finding an evaluation tool that allows us distinct insights into students' knowledge acquisition, we found, that PTP can be successfully adapted as a method to periodontal teaching in the German university setting. The dentistry programme at Kiel University is structured according to the German licencing regulations for dentists, a framework for the structure of dentistry study programmes nationwide. These include, that the subject periodontology is taught in the clinical part—the second half‐of the curriculum in the context of the subject group of conservative dentistry. Within this juristic framework, faculties can decide in which semester which lecture is situated; in Kiel, a longitudinal approach as described above is followed. This allowed the developed PTP four measurement points during the clinical part of our curriculum. In detail, we found a growth curve in the first three semesters with a ceiling effect, as is also to be observed in other studies with similar context in medicine [7, 17, 30, 33]. Verhoeven et al. [4] found a linear growth of knowledge but a slowing growth rate when looking at single subcategories, which goes in line with our findings. Assuming that the students had little to no periodontal knowledge at the start of the periodontal courses, the courses in the first semester had the greatest impact on the mean score. Between the first and second clinical semesters, the mean score grew almost 20%, whereas between the second and third semesters, the observed growth was slightly lower. An explanation for that could be that the basics imparted in the earlier semesters were easier to teach as well as to remember compared to specific details taught in the later semesters. Cecilio‐Fernandes et al. specifically found students in later semesters improving at answering more complex clinical questions [34]. The decrease in the current investigation could be explained by a gap of 1 year between the end of specific periodontal teaching and the final PTP (end of curricula), also enhanced by the higher semester internal homogeneity expressed by a slightly decreasing standard deviation towards the end of the programme. On the other side, in the first clinical semester, there were some outbreaking scores, indicating a greater span of knowledge between the participating students. This may occur because of the different knowledge levels of students at the beginning of our clinical courses (e.g., previous experience or professional education in dentistry). The constant increase of scores in our curriculum for the PTP indicated high validity [12] and acceptable reliability [35] due to the small cohort sizes [17, 33, 36]. Overall, the development of the achieved score by students of each semester showed a growth of knowledge and knowledge application [37]. It indicated that long‐term knowledge retention, assumed to be one advantage of PT compared to traditional examinations in the literature [8], was encouraged by this test format as our dental students revised not only the most recent topics in preparation for the test.

Beside the present data, the first challenges we had to solve were creating a blueprint representing the periodontal expertise a graduated dentist should know. A difficulty has been the limit of 30 questions, thus not every blueprint area is represented in the test. Blueprints are supposed to have a positive effect on tests comparability and our results showed satisfying homogeneity of item difficulties within test occasions. Significantly better results on test occasion four might be caused by students' accustoming to the new test format. Further challenges were (1) distinguishing the areas in the blueprint when an item over lapses two or more areas. As (2) it came out to be hard writing significant questions for all specific blueprint areas without getting repetitive as periodontology plays a comparably small role in the German dental curriculum at the current time. Point (3.) has to be seen as a limitation, as no structured item revision board of PT's items [15] has been formed due to lack of personal capacities. Still, we discussed each question in a group with all experts before the tests. However, to accomplish (4.) writing questions in the dental context, the medicine, on diagnosis had to be adapted to more technically based content [16] in the subject of periodontology where a profound knowledge about aetiology and various technically complex treatment methods are clinically relevant. The question bank should be expanded continuously to prevent students from remembering items; however, this is time and resource consuming [5, 10, 11, 12, 16, 38]. Regarding only one subject within the whole dental curriculum, those efforts were reduced. With limited personal and technical resources, direct feedback to all students has not been possible so far, but would be desirable to enhance the test's direct benefits in a formative setting [39].

The use of the ‘I don't know’ option was new to our dental students. It showed a high on first clinical semester whilst decreasingly used by the other semesters, comparable to previously published studies [17, 40]. This might have occurred because dental students get to treat patients early, in our curricula in the second clinical semester. At this point, students are being trained to make quick decisions as they are confronted with more complex clinical problems on a daily basis. This development might also be the reason why the most unanswered questions occur after the second clinical semester; the students needed more time than was provided for making their decisions. Several studies observed a significant increase in correct answers along with their clinical experience in placement during the curriculum [6, 17, 33]. Items answered with ‘I don't know’ by last years' students might indicate that the subject is not covered enough during the curriculum or the wording is confusing [4]. On the other hand, the option is a legitimate answer especially for first and second semester students, as they cannot yet answer all questions from the imparted knowledge alone. Apparent ‘gaps of knowledge’ thus have to be analysed individually afterward especially by the faculty staff. Another way to improve PTP is to offer more detailed feedback for students in order to help them identify their strengths and weaknesses [25].

All in all, we found a PT has the potential to evaluate the dental curriculum in all areas [16, 17, 33]. Since our PTP was curriculum independent, it also showed potential to be used at other faculties and therefore reduce costs and effort [7, 25, 41]. It is therefore also interesting to see the integration of the PTP in the context of the new licencing regulations in Germany from 2019 (^2^). This is especially interesting for the evaluation of the newly implemented so‐called integrated clinical courses, combining conservative, periodontal and prosthetic dentistry, closing the mentioned gap in fourth and fifth semester without any periodontal teaching. Additionally, in an international context: For periodontology, the consensus workshop of the European Federation of Periodontology (EFP) in 2009 developed recommendations on appropriate competencies, teaching and evaluation methods in basic periodontal training [42]. However, a survey conducted in 2018 showed that the teaching methods of the individual schools vary greatly and efforts should be made to improve and harmonise undergraduate periodontal education programmes [43]. A recently published systematic review by Preshaw et al. collected innovative periodontal teaching approaches and sees a future in so‐called blended approaches with both face‐to‐face and digital aspects. The implementation of these however should be assessed ongoingly [44] which can be performed by the PTP.

Regarding the restructure of the dental curriculum at Kiel University, it should be minded to spread courses longitudinally rather than having gaps that lead into knowledge stagnation or even potential loss. So far, the test only investigated the current educational setting and hinted towards potential quality issues in the teaching. However, there is potential to observe changes being made to the periodontal curriculum in the coming test occasions. Future PTP results will show if the changes have been made successfully, for example, via analysing students' progress in the blueprint subcategories [6].

### 4.1 Strengths and Weaknesses

Some considerations have to be made when interpreting the results that may bias them. Firstly, the cohorts were quite small in comparison to the medical cohort. During the observation period of the study, classes were mainly held online due to the pandemic. Therefore, it must be assumed that not all students watched all screencast lectures. Nevertheless, some of the cohorts (A–C) received face‐to‐face teaching due to the dynamic nature of the pandemic and the constantly changing regulations on how teaching is possible. We know that our dental students showed a normal or mild psychological impact during the COVID‐19 pandemic [45] which leads to the conclusion that the pandemic itself had only a minor influence on the presented study. The results of the well‐established Progress Test Medicine even showed a growth of knowledge gain during the pandemic [46]. Additionally, no semester cohort participated at all four semester levels observed in this study. The poorer participation of students in their final year goes in line with the optional participation and scores might be biassed by this student cohort being a self‐selecting group. Nevertheless, there seemed to be an interest in using the PTP as a ‘final rehearsal’ before their final exam. In those cohorts where it has been the second time participating in the PTP non‐mandatory participation ranged from 52% to 68%.

The test setting using the online platform has proven to be convenient for both students and faculty and also allowed the use of images [16]. Bias might be caused by letting students do the test at home, so cheating could not be controlled, for example screenshotting questions and passing them on. Regardless, it was discouraged by the formative character of the test, by a large and continuously extended question bank and compounded by the tight time limit and other restrictions stated above. Nevertheless, some students can handle the stress of time limitation better than others, which might also bias the results. Although a time limit was specified for the participants in all tests, it was found that the majority of all participants (84.64%) were able to answer the questions in the time available. Thus, further evaluations should also be carried out with regard to this aspect. The questions themselves have been written and revised carefully to avoid bias caused by cuing within the distractors. This is achieved by the already mentioned multi‐stage control process ensuring adherence to the guidelines [23, 24, 25, 26]. As there were little questions reused (< 10%), we would expect a minor impact to screenshot passed down by students on the test results. The formative setting is found less stressful and therefore increases acceptance [47]. In detail, it can foster academic motivation and self‐regulation skills whilst reducing anxiety [48], which might reduce the bias of panic and blackouts during tests.

Another weakness of the study is that the scoring result was used as a direct indicator of students' knowledge. According to Dijksterhuis et al. [13] and Kanzow et al. [49] scores cannot be equated with test takers' so‐called true knowledge as there is the possibility of guessing, especially if it is not penalised by subtraction of points. The probability of right guesses in relation to the number of distractors and under consideration of partial knowledge (‘informed guessing’) has to be taken into account. Unrelated to the chosen scoring method, they however showed a linear correlation between score and knowledge. Accordingly, this allows resumes on students' knowledge. A statistically calculated test equation to compensate for the difference between mean item difficulty benefits the comparability between test occasions. There are several options to perform it, while still being challenging for PTs with a rather small number of participants [50]. In this context over observing general growth patterns and therefore overlaying the test occasions, it is less crucial compared to when tracking individual scores longitudinally.

## 5 Conclusion

This is the first reported implementation of progress testing in periodontology inside an undergraduate dental curriculum in Germany. Within the limitations of the study, the results indicated PTP as a valid tool to evaluate students' learning longitudinally; however, establishing PTP as a standardised learning assessment is a time and capacity‐consuming procedure. At our university, PTP will be continued and accompany the introduction of the new licencing regulations in periodontology.

## Author Contributions

A.L.K. and C.G. developed together with author C.E.D. the idea of the investigation and performed the statistical analyses. M.C. and M.M. performed the data acquisition, and together with J.C. interpreted the data. A.L.K., C.G. and M.C. drafted and critically revised the manuscript. All authors read and approved the final manuscript.

## Ethics Statement

All procedures were performed in accordance with the ethical standards of the institutional and national research committees (Kiel IRB: D452/18), as well as the 1964 Declaration of Helsinki and their more recent versions, and were approved by the ethics committee of the medical faculty of the Christian‐Albrechts‐University of Kiel. Before starting the study, verbal and written information about the study protocol was given to the participants and they had to provide written informed consent before enrollment.

## Conflicts of Interest

The authors declare no conflicts of interest.

## Data Availability

The data that support the findings of this study are available from the corresponding author upon reasonable request.

## Endnotes

^1^

https://progress‐test‐medizin.charite.de/fileadmin/user_upload/microsites/sonstige/ptm/Downloads/FragenleitlinienPTM.pdf, Last accessed: 16.03.2024.

^2^

https://www.gesetze‐im‐internet.de/zappro/BJNR093310019.html., Last accessed: 16.03.2024.
